# Monozygotic Twins Suffering From Sodium Taurocholate Cotransporting Polypeptide Deficiency: A Case Report

**DOI:** 10.3389/fped.2018.00354

**Published:** 2018-11-20

**Authors:** Hui-Jun Tan, Mei Deng, Jian-Wu Qiu, Jun-Feng Wu, Yuan-Zong Song

**Affiliations:** ^1^Department of Pediatrics, The First Affiliated Hospital of Jinan University, Guangzhou, China; ^2^Department of Infectious Diseases, Quanzhou Women's and Children's Hospital, Quanzhou, China

**Keywords:** sodium taurocholate cotransporting polypeptide deficiency, *SLC10A1*, bile acid, hypercholanemia, cholestasis

## Abstract

Sodium taurocholate cotransporting polypeptide (NTCP) is a carrier protein encoded by the human *SLC10A1* gene, acting as the principal transporter of conjugated bile salts from the plasma into hepatocytes. Although NTCP was cloned as early as in 1994 and its function has been studied extensively, clinical description of NTCP deficiency remains rather limited thus far. The patients in this paper were 2 female monozygotic twins, who were referred to our hospital at the age 2 years with the complaint of persistently-raised total bile acids (TBA) for 21 months. At age 3 months, they were both diagnosed to have cholestatic liver disease due to raised serum TBA and direct bilirubin (DBIL) with the fraction >20% of the elevated total bilirubin (TBIL). Thereafter, their jaundice subsided and the DBIL levels recovered gradually, while serum TBA remained raised persistently. In view of their refractory hypercholanemia but negative symptoms and signs, *SLC10A1* genetic analysis was performed for all family members to evaluate the possibility of NTCP deficiency. As a result, the twins were both homozygotes, while the parents, carriers, of the reportedly pathogenic variant c.800C>T (p.Ser267Phe). These findings suggested that NTCP deficiency may be a unique genetic factor causing transient cholestasis in early infancy, as well as, persistent hypercholanemia in pediatric patients.

## Introduction

Deficiency of sodium taurocholate cotransporting polypeptide (NTCP) is an inborn error of bile acid metabolism caused by biallelic *SLC10A1* variants, which impairs the NTCP function as the primary transporter of conjugated bile salts from the plasma into hepatocytes (1–4). NTCP was cloned as early as in the year 1994 (5), and thereafter, its function has been studied extensively (6, 7), while a number of *SLC10A1* genetic variants have been identified in humans (1, 7, 8). However, the first patient with NTCP deficiency was just reported very recently by Vaz et al. (9). Since then, several papers about patients with NTCP deficiency have been published (4, 10–14), but the patient number was limited and the laboratory and clinical features of this new disorder still remained open for further investigation.

Herein, we reported two monozygotic twins suffering from NTCP deficiency, who presented with persistent hypercholanemia and transient cholestatic jaundice in their early infancy.

## Case description

*Patient 1*. A 2-years-old female infant was referred to the First Affiliated Hospital, Jinan University, due to elevated serum total bile acids (TBA) discovered for 21 months. At the age 3 months, she was admitted to Women and Children's Hospital of Quanzhou because of jaundice for 2 months. Laboratory test revealed that the serum levels of TBA, direct bilirubin (DBIL), aspartate transaminase (AST) and alanine transaminase (ALT) were all elevated (Table 1), and she was thus diagnosed to have cholestatic liver disease. After being treated with intravenous reduced glutathione and ademetionine-1,4-butanedisulfonate for 9 days, which were commonly used in cholestatic patients with elevated alanine transaminase levels, her jaundice was alleviated, but the liver function indices remained abnormal (Table 1). Then oral ursodesoxycholic acid was given and she was discharged at the age 3.3 months. During the subsequent follow-up over 20 months, she showed normal anthropometric and neurobehavioral development without abnormal appearance, and her cholestasis resolved completely since the age 7 months. However, persistently raised TBA levels were observed on repeated biochemical analyses (Table 1). So she was referred to our hospital for further investigation and management when aged 2 years.

**Table 1:** Biochemical indices of the monozygotic twins and their parents.

**Indices (reference range)**		**Patient 1/Patient 2**											**Parents**	
	**3M^a^**	**3.3M**	**7M**	**8M**	**10M**	**12.3M**	**14.2M**	**15.7M**	**18.3M**	**21.6M**	**24M^b^**	**27M**	**Father**	**Mother**
TBIL (2–19 μmol/l)	106.5/73.1	34.9/23.3	11.6/12.7	6.9/7.8	4.4/3.9	4.2/3.9	4.3/3.7	3.8/4.5	3.4/4.0	7.4/7.4	5.2/4.8	10.2/10.1	11.8	7.7
DBIL (0–6 μmol/l)	67.6/45.6	21.7/13.8	2.8/3.5	1.6/2.1	1.5/1.1	1.2/1.3	1.2/1.0	1.1/1.2	1.1/1.3	2.2/1.8	1.6/1.3	2.9/3.2	3.5	2.6
IBIL(2.56–20.9 μmol/l)	38.9/27.5	13.2/9.5	8.8/9.2	5.3/5.7	2.9/2.8	3.0/2.6	3.1/2.7	2.7/3.3	2.3/2.7	5.2/5.6	3.6/3.5	7.3/6.9	8.3	5.1
TBA (0–10 μmol/l)	221/210.8	223/239.1	170.1/144	171.2/138.9	211.4/225	107.4/139.1	187.1/123.2	179.9/276.1	103.0/184.1	104.8/105.4	173.8/198.3	162.6/118	18.5	5.1
ALT (5–40 U/l)	100/95	122/96	68/49	34/25	142/38	21/23	25/24	18/21	20/17	22/17	15/13	19/16	15	19
AST (5–40 U/l)	114/106	112/90	68/56	52/51	76/55	47/50	51/42	36/41	40/45	43/45	35/38	40/37	16	18
GGT (8–50 U/l)	110/112	63/46	17/16	10/9	10/9	7/7	9/8	8/9	14/9	9/12	8/9	7/8	18	12
ALP (20–500 U/l)	391/34.5	385/326	172/164	190/192	184/199	286/277	241/240	179/192	205/245	237/264	165/170	157/172	79	49
TP (60.0–83.0 g/L)	41/40.5	–	57.4/54.3	59.4/58.8	63.6/3.5	62.4/66.9	63.7/61.3	66.4/65.5	62.8/70.2	66/67.8	66.4/70.1	66.8/66.3	70.8	67.3
ALB (35.0–55.0 g/L)	–	–	40.3/38.9	40/40.1	40.5/41.8	42.9/44.4	43.3/41.7	41.3/41.1	39.3/42.7	43.5/44.4	43.2/44.2	43.3/43.4	48.8	45.3

a *First visiting to the local hospital*.

b *Referral to our clinic*.

*TBIL, total bilirubin; DBIL, direct bilirubin; IBIL, indirect bilirubin; TBA, total bile acids; ALT, alanine transaminase; AST, aspartate transaminase; GGT, gamma-glutamyl transpeptidase; ALP, alkaline phosphatase; ALB, albumin*.

The patient was the elder sister of two monochorionic diamniotic twins who was delivered by cesarean section at the gestation age of 37 weeks and 2 days with the birth weight 2.25 kg and body length 45.0 cm. Her father is a hepatitis B virus (HBV) carrier who was clinically healthy but with slightly elevated serum TBA level on biochemistry analysis, and her mother was physically and biochemically healthy (Table 1). There was no family history of any genetic disease.

Physical examination revealed a body weight of 12.0 kg, height 83.5 cm and head circumference 46.0 cm. No jaundice was observed in the skin and sclera. No stridor, crackles or crepitus was heard in the two lungs, and the heart sound was normal without any murmurs. There was no abdominal distention, and the liver and spleen were non-palpable. Physiological reflexes were normal and no pathological reflexes could be found on nervous system examination. On biochemical analysis, the TBA level reached 173.8 μmol/L (0–10 μmol/L) as other indices were normal (Table 1).

*Patient 2* was the younger sister of patient 1, who also experienced transient cholestatic jaundice in early infancy and was referred to our hospital with the same complaint of persistently elevated serum TBA levels. As the younger one of two monozygotic twins, her birth weight was 2.30 kg and body length, 46.0 cm. On physical examination at referral, the body weight was 13.0 kg, height 85.0 cm, and head circumference 46.0 cm. No jaundiced skin or sclera was observed. Examinations of the lungs, the heart, the abdomen and nervous system were all normal. Her TBA level was 198.3 μmol/L (0–10 μmol/L), with otherwise normal biochemistry indices (Table 1).

**Figure 1:**
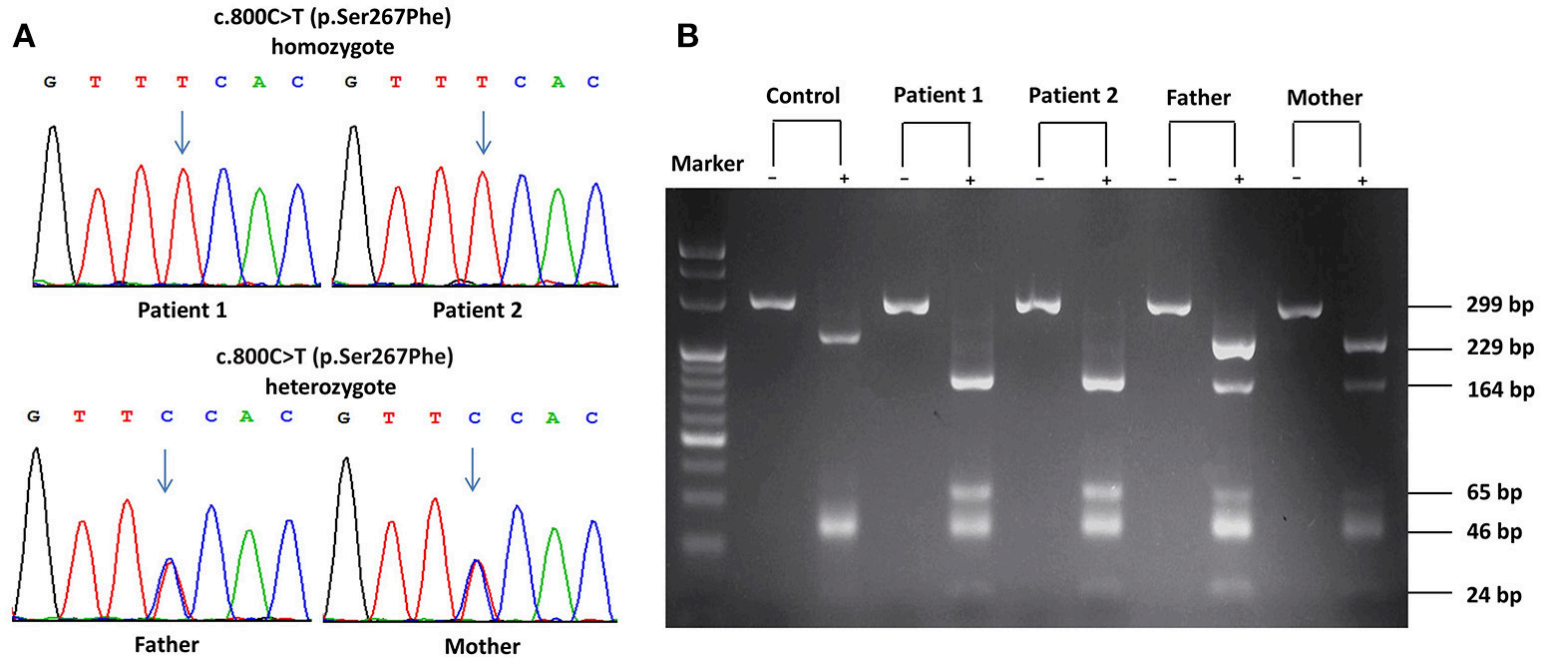
*SLC10A1* genotypes of all family members on Sanger sequencing and electrophoresis of the PCR-RFLP products. **(A)** The two patients were both homozygotes while their parents, carriers, of the c.800C>T (p.Ser267Phe) variant. **(B)** PCR-RFLP analysis confirmed the *SLC10A1* genotypic findings as in **(A)**. In **(B)**, the symbols “+” and “–” denoted with and without digestion of the restriction enzyme *Hph*I, respectively.

Considering their refractory hypercholanemia whist lack of clinical symptoms and signs on physical examination, NTCP deficiency was highly suspected and hence Sanger sequencing of the *SLC10A1* gene was carried out for all family members. As a result, the twins were both homozygotes of the variant c.800C>T (p.Ser267Phe), and their parents were both carriers of the same variant (Figure 1A). Electrophoresis of the PCR-RFLP products showed that the twins both harbored two additional bands of 164 bp and 65 bp rising from the aforementioned *SLC10A1* variant, which further confirmed the *SLC10A1* genotypes of all family members (Figure 1B).

NTCP deficiency was thus definitely diagnosed, and the twins were followed-up in the local hospital, and no specific medication was given. Thus far, the twins were both healthy but the serum TBA levels were still raised (Table 1), and their long-term outcomes needed to be observed.

## Discussion

In this paper, *SLC10A1* genetic analysis provided reliable evidences for the definite diagnosis of NTCP deficiency for the monozygotic twins. The p.Ser267Phe variant in *SLC10A1* gene had proven pathogenic by functional, bioinformatic, and clinical evidences (4, 10–12). Of note, this *SLC10A1* variant was prevalent in East Asian population, with an allele frequency of 8% in Southern Han Chinese, 12% in Chinese Dai and 11% in Vietnam (http://grch37.ensembl.org/Homo_sapiens/Variation/Population?db=core;r=14:70244693-70245693;v=rs2296651;vdb=variation;vf=1699086) suggesting that this type of hypercholanemia might affect 0.64% of the Southern Han, 1.44% of the Dai Chinese, and 1.21% of the Vietnamese population (11). In addition, other non-synonymous *SLC10A1* variants have been identified previously in different ethnic populations as well, such as p.Ile223Thr and p.Ala64Thr, with an allele frequency of 5% in African Americans and 1% in Korean, respectively (1, 7). Therefore, although patients of NTCP deficiency were rarely reported in the past over 20 years, NTCP deficiency may not be rare worldwide, especially in East Asian population.

Persistent hypercholanemia was the prominent feature of the patients with NTCP deficiency in this paper. Bile acids are synthesized from cholesterol in the liver, secreted into bile which is stored in the gallbladder, and then enter the small intestine during a meal. The majority (>90%) of bile acids are reabsorbed from the intestine and returned to the liver via the portal venous circulation. This circling of bile salts is well-known as enterohepatic circulation (15). NTCP plays a key role in the enterohepatic circulation of bile salts as the major transporter of conjugated bile salts from the plasma compartment into the hepatocyte (5). The p.Ser267Phe variant had been reported to result in an almost complete loss of the function for bile acid uptake, rendering NTCP without taurocholate transporting activity (6, 7, 10). Although the hepatocyte sinusoidal membrane also expresses other sodium-independent bile acid transporters, these transporters play limited roles in bile acid clearance (16). As such, it was not strange for the twins in this paper to present with refractory hypercholanemia.

Of particular note, the father of the twins, who was a carrier of the p.Ser267Phe variant and HBV as well, also exhibited slight hypercholanemia (Table 1). Since the reported patients with NTCP deficiency thus far were all compound heterozygotes or homozygotes of *SLC10A1* biallelic variants (4, 9–14), indicating an autosomal recessive disorder, the slightly elevated TBA level in the father could not be explained by his carrier status of the p.Ser267Phe variant. Recently, Yan et al. found that NTCP also functions as a cellular receptor for viral entry of HBV through a specific interaction between NTCP and the pre-S1 domain of HBV large envelope protein (17), and showed that pre-S1 lipopeptide binding blocked the physiological function of NTCP in bile salt transport (6). As such, it is reasonable to speculate that the HBV carrier status of the father might affect the NTCP function to uptake bile acid at the basolateral membrane of the hepatocyte, being responsible for his slight hypercholanemia, as shown in Table 1.

Besides the persistent hypercholanemia, the elevated DBIL (67.6 μmol/l and 45.6 μmol/l) fraction >20% of TBIL (106.5 μmol/l and 73.1 μmol/l) in the twins at 3 months (Table 1) clearly indicated cholestatic jaundice in early infancy (18), and lent support to the concept that NTCP deficiency might work as one of the contributing factors that affect the bilirubin homeostasis, particularly in early infants who have very rich bilirubin sources but immature liver function to uptake, conjugate, and excrete bilirubin (19). Actually, hepatic uptake of bile salts is mediated by sodium-dependent and sodium-independent transporters (20). Sodium-independent uptake of bile salts is principally accomplished by the activity of multiple organic anion transporting polypeptides 1B1 and 1B3(OATP1B1/1B3), which work as an dimer protein to uptake plasma bilirubin, especially the direct bilirubin, as well as, bile acids into the hepatocyte (21, 22). NTCP is the primary sodium-dependent transporter of conjugated bile salts from the plasma compartment into the hepatocyte (5). Therefore, the elevated plasma bile acids caused by the deficiency of NTCP might increase the burden of the OATP1B1/1B3 to uptake bile acids into the hepatocyte, competitively inhibit its' function to uptake bilirubin, and result in secondary elevation of bilirubin, especially direct bilirubin, giving rise to cholestatic jaundice in infants as in the twins in this study, as well as, in NTCP-deficient patients reported previously (4, 10, 14).

## Conclusion

In summary, this study reported the clinical and genetic findings of two monozygotic twins with NTCP deficiency. Our findings lent support to the important role of NTCP in bile salt homeostasis, and suggested that NTCP deficiency may be a unique genetic factor causing cholestasis in early infancy, as well as, persistent hypercholanemia at pediatric age.

## Informed consent

The authors declare that this study was performed after written informed consent had been obtained from the parents of the twins, which permitted publication of this case report.

## Ethics statement

This study has been approved by the Committee for Medical Ethics, the First Affiliated Hospital, Jinan University.

## Author contributions

H-JT performed data collection and drafted the initial manuscript. Y-ZS conceptualized and designed the study, critically reviewed, and revised the manuscript. MD and J-WQ carried out the genetic analyses and reviewed the manuscript. J-FW treated and followed up the patients and reviewed the manuscript. All authors contributed to manuscript revision, read, and approved the submitted version.

### Conflict of interest statement

The authors declare that the research was conducted in the absence of any commercial or financial relationships that could be construed as a potential conflict of interest.

## References

[1] Pan W Song IS Shin HJ Kim MH Choi YL Lim SJ. Genetic polymorphisms in Na+-taurocholate co-transporting polypeptide (NTCP) and ileal apical sodium-dependent bile acid transporter (ASBT) and ethnic comparisons of functional variants of NTCP among Asian populations. Xenobiotica (2011) 41:501–10. 10.3109/00498254.2011.555567 21341987

[2] Hagenbuch B Dawson P. The sodium bile salt cotransport family SLC10. Pflugers Arch. (2004) 447:566–70. 10.1007/s00424-003-1130-z 12851823

[3] Anwer MS Stieger B. Sodium-dependent bile salt transporters of the SLC10A transporter family: more than solute transporters. Pflugers Arch. (2014) 466:77–89. 10.1007/s00424-013-1367-0 24196564 PMC3877701

[4] Song YZ Deng M. Sodium taurocholate cotransporting polypeptide deficiency manifesting as cholestatic jaundice in early infancy: a complicated case study. Zhongguo Dang Dai Er Ke Za Zhi (2017) 19:350–4. 10.7499/j.issn.1008-8830.2017.03.020 28302211 PMC7390148

[5] Hagenbuch B Meier PJ. Molecular cloning, chromosomal localization, and functional characterization of a human liver Na+/bile acid cotransporter. J Clin Invest. (1994) 93:1326–31. 10.1172/JCI117091 8132774 PMC294097

[6] Yan H Peng B Liu Y Xu G He W Ren B. Viral entry of hepatitis B and D viruses and bile salts transportation share common molecular determinants on sodium taurocholate cotransporting polypeptide. J Virol. (2014) 88:3273–84. 10.1128/JVI.03478-13 24390325 PMC3957944

[7] Ho RH Leake BF Roberts RL Lee W Kim RB. Ethnicity-dependent polymorphism in Na+-taurocholate cotransporting polypeptide (SLC10A1) reveals a domain critical for bile acid substrate recognition. J Biol Chem. (2004) 279:7213–22. 10.1074/jbc.M305782200 14660639

[8] Saito S Iida A Sekine A Oqawa C Kawauchi S Higuchi S. Catalog of 238 variations among six human genes encoding solute carriers (hSLCs) in the Japanese population. J Hum Genet. (2002)47:576–84. 10.1007/s100380200088 12436193

[9] Vaz FM Paulusma CC Huidekoper H de Ru M Lim C Koster J. Sodium taurocholate cotransporting polypeptide (SLC10A1) deficiency: conjugated hypercholanemia without a clear clinical phenotype. Hepatology (2015) 61:260–7. 10.1002/hep.27240 24867799

[10] Deng M Mao M Guo L Chen FP Wen WR Song YZ. Clinical and molecular study of a pediatric patient with sodium taurocholate cotransporting polypeptide deficiency. Exp Ther Med. (2016) 12:3294–300. 10.3892/etm.2016.3752 27882152 PMC5103782

[11] Liu R Chen C Xia X Liao Q Wang Q Newcombe PJ. Homozygous p.Ser267Phe in SLC10A1 is associated with a new type of hypercholanemia and implications for personalized medicine. Sci Rep. (2017) 7:9214. 10.1038/s41598-017-07012-2 28835676 PMC5569087

[12] Van Herpe F Waterham HR Adam CJ Mannens M Bikker H Vaz FM. NTCP deficiency and persistently raised bile salts: an adult case. J Inherit Metab Dis. (2017) 40:313–5. 10.1007/s10545-017-0031-9 28283843

[13] Qiu JW Deng M Cheng Y Atif RM Lin WX Song YZ Sodium taurocholate cotransporting polypeptide (NTCP) defciency: identifcation of a novel SLC10A1 mutation in two unrelated infants presenting with neonatal indirect hyperbilirubinemia and remarkable hypercholanemia. J Oncotarget (2017) 8:106598–607. 10.18632/oncotarget.22503 PMC5739759 29290974

[14] Li H Qiu JW Lin GZ Deng M Lin WX Song YZ. Clinical and genetic analysis of a pediatric patient with sodium taurocholate cotransporting polypeptide deficiency. Zhongguo Dang Dai Er Ke Za Zhi (2018) 20:279–84. 10.7499/j.issn.1008-8830.2018.04.005 29658451 PMC7390035

[15] Dawson PA Role of the intestinal bile acid transporters in bile acid and drug disposition. Handb Exp Pharmacol. (2011) 201:169–203. 10.1007/978-3-642-14541-4_4 PMC3249407 21103970

[16] Karpen SJ Dawson PA. Not all (bile acids) who wander are lost: the frst report of a patient with an isolated NTCP defect. Hepatology (2015) 61:24–7. 10.1002/hep.27294 24995605 PMC4280297

[17] Yan H Zhong G Xu G He W Jing Z Li W. Sodium taurocholate cotransporting polypeptide is a functional receptor for human hepatitis B and D virus. Elife (2012) 1:e00049. 10.7554/eLife.00049 23150796 PMC3485615

[18] Andrianov MG Azzam RK. Cholestasis in infancy. Pediatr Ann. (2016) 45:e414–9. 10.3928/19382359-20161118-01 27975109

[19] Grijalva J Vakili K. Neonatal liver physiology. Semin Pediatr Surg. (2013) 22:185–9. 10.1053/j.sempedsurg.2013.10.006 24331092

[20] Van Dyke RW Stephens JE Scharschmidt BF. Bile acid transport in cultured rat hepatocytes. Am J Physiol. (1982) 243:G484–92. 7149031 10.1152/ajpgi.1982.243.6.G484

[21] Hagenbuch B Meier PJ. The superfamily of organic anion transporting polypeptides. Biochim Biophys Acta (2003) 1609:1–18. 10.1016/S0005-2736(02)00633-8 12507753

[22] Erlinger S Arias IM Dhumeaux D. Inherited disorders of bilirubin transport and conjugation: new insights into molecular mechanisms and consequences. Gastroenterology (2014) 146:1625–38. 10.1053/j.gastro.2014.03.047 24704527

